# Position in Labor

**Published:** 1873-10

**Authors:** J. W. Smith

**Affiliations:** Charles City, Iowa


### SELECTIONS.

### POSITION IN LABOR.

READ BEFORE THE IOWA STATE MEDICAL SOCIETY, MAY 28, 1873,
BY J. W. SMITH, M. D., CHARLES CITY, IOWA.

More than thirteen years ago, an accidental circumstance directed my attention to the investigation of position in labor; and profoundly impressed with the necessity of more definite views among accouchers as to the best position, and the ease and certainty with which it can usually be ascertained, I feel it a duty to make known my investigations.

Opportunities for observation in private practice are not equal to a large lying-in-hospital, but they have been sufficient to convince me that there is a more excellent way than that of guess-work, or convenience, as to the position of a paturient woman. Such experience has also taught me that, in the majority of cases, the proper position can be determined with almost mathematical accuracy, and as one result, the duration of the labor will often be materially shortened.

Text-books and obstetric teachings—at least all within my acquaintance—have little that is satisfactory and definite upon the subject. Such facts prove (1) that position is of minor importance, or else (2) it is not well understood. This state of things is unfavorable to obstetric science, unsatisfactory to the young practitioner, and most unfortunate to suffering woman.

Cannot the accoucher be furnished with unfailing directions, in each particular case, as to the position that will cause the labor to be the safest, easiest and quickest possible? I believe that not only possible, but within the easy comprehension of every physician of ordinary capacity, and who carefully studies the subject.

Doubtless many cases do equally well when the position is decided wholly by accidental circumstances. Among the things that are important at the beginning of every labor may be mentioned the proper evacuation of the bowels—a free enema of tepid water, or of salt or soap in water, being advantageous in the majority of cases. The bladder should be frequently evacuated, the clothing loose, and the under-garments so arranged as not to require change after delivery; the feet kept warm, the head cool, the room quiet and well ventilated. A bed—not of feathers—or a broad lounge should be in readiness.

During the early stage of labor no restriction of movement should be attempted; but moderate exercise, especially walking, should be encouraged, and insisted upon in some cases, until it becomes painful or uncomfortable.

An early examination is to be recommended. When the os is so high as to be reached with difficulty, it is not possible, in all cases, to decide as to the precise presentation, and, of course, not as to the best future position; but as the case progresses, and usually long before the rupture of the membrane, it is easy to decide as to the proper position. A patient's choice is often wrong, hence the need of deciding for her.

The philosophy of position is based upon gravity and muscular action, including contraction and relaxation. The key-note, or axiom, so to speak, of correct position, is the fact that the fundus of of the gravid uterus, in its normal state, is *movable* to a certain extent in nearly all cases when not obstructed. The field for the application of this principle is almost unlimited, and will vary to some extent in every individual case.

### HOW TO DETERMINE THE PROPER POSITION.

The directions how to decide as to the proper position had better be explicit; and to save time and space we will avoid all technicalities and proceed at once to explain how it is possible to decide upon the position in which the parturient powers will most quickly, easily and safely overcome the natural resistance. External abdominal examination aids in diagnosis, and should not be omitted in doubtful cases, but is not usually as reliable as that per vaginum.

Suppose that we cannot tell the exact presentation, or that we can, and in either case that the presenting part is found to impinge or press strongly upon or towards the *left* side of the pelvis, and even while the os does sometimes point, so to speak, to the *right* side of median line of the pelvis. These two things occurring together may be said to be contradictory, and they do appear so at first observation—that is, the os inclined to the right, and the bulk of the fœtus to the left side of the pelvis. To decide accuratey, before or after the full dilatation of the uterus, a gentle upward pressure of the fœtus by the finger, between pains, will often be necessary. The finger can then be passed from side to side, and thus determine

*which side of the pelvis is most filled or pressed upon by the presenting part.* If, as supposed, the presenting portion presses most directly upon, or to the left side of, the pelvis, and, as stated, independently of the position of the os, that side—upon the left—is the position in which it will uniformly be found in practice best to place the patient; if to the right, the position should be upon that side. This is very easy to remember.

When placed upon the side, as indicated, it will often make all difference *how* the woman is placed—at least during a portion of the time—occasional changes of position being admissable, and sometimes necessary in the earlier stages to prevent too great fatigue or disgust with any one position. The woman's head should be low, the fundus moving more freely by gravity when it is so ; the thighs and knees fixed; the feet supported, if preferred ; and the arm that is under side placed either wholly back of the body, or, which is often easier, the elbow sharply flexed, and only the hand allowed to rest in front of the chest. She will then be partly upon her face, as in the position for using Sim's speculum, and cannot then turn her back, as otherwise she would be likely to do, and thus defeat the full advantage of position. The knees, in the latter stages of labor, had better be supported by an assistant, or by a pillow or quilt firmly rolled together and placed between them.

In many cases of tedious labor, after the *proper* position is assumed, such position, and a single pain or two, if strong, will often bring the fœtus into the proper axis of the pelvis, and thus rapidly complete the labor. When the effect is not so rapid, and some other position is preferred, it can be assumed without detriment, after the cause of delay has been overcome.

### ABDOMINAL SUPPORT.

In case of obliquity, pendulous abdomen, uterine inertia, etc., a wide bandage or support is often a great help, and sometimes necessary, in addition to the best position. It can be readily extemporized, and while it may not be as convenient as some specially designed, with me it has answered admirably. It should be about two yards in length, from twelve to eighteen inches wide, and made of strong material. Two towels can be sewed together, or a sheet torn lengthwise and doubled once in the same direction.

### HOW TO USE IT.

In lateral obliquity it is always best, and sometimes in other cases, to place the woman upon her side, as already described, with the centre of the bandage passed evenly across the abdominal prominence, with one end carried under and the other over, and both behind the patient, so that the two ends can be firmly grasped together by one or both hands, usually best by the physician, and steady pressure can then be made across and around the abdomen during each pain. To make such pressure reliable, will require the counter support of the back at the same time, and this can often be made the most certainly and easily by the foot or knee of the person holding the bandage, a pillow or other soft support being first placed against the back, and, if room, the person sitting upon or standing by the side of the bed, while the abdominal pressure is applied by the bandage. The two forces can thus be made to act together, and keep the woman in the desired position, while varying the amount and direction of the support, as is indicated. The bandage should be seized near the ends, to prevent too great compression of the sides of the woman. To aid sufficiently in some cases, may require the application of the bandage continuously for a length of time, or only occasionally and during a few pains. Posterior obliquity, as in pendulous abdomen, can sometimes be corrected by dorsal position, elevation of the hips, and external pressure by the hands; but experience teaches that the side position and bandage is often most effectual in those cases. Uterine inertia can sometimes be overcome by elevating the shoulders and pressure with the hands, but the kneeling or standing posture alone is sometimes sufficient and even preferable, the patient to be supported under the arms by two assistants during each pain.

### SUMMARY OF DIRECTIONS.

To epitomize or briefly recapitulate the foregoing directions: Most cases have a lateral obliquity or pressure, so to speak, and if the presenting part crowds upon, or most nearly fills one side of the pelvis, then upon that side is the proper position. If towards the hollow of the sacrum, place the woman upon her back, elevate the hips, and, if necessary, use external pressure. If towards the sacrum, and one side, better place her upon that side, observing the arm is in position ; and, if necessary, apply the bandage and support the back. If upon or towards the symphysis pubis, place her in a kneeling and almost horizontal posture, and, if necessary, upon the knees and face for a short time.

Formerly I was partial to the position upon the back, as I thought it easy and convenient, but observation convinced me that the large majority of cases do best upon one side or the other. It seems to most readily correct and overcome the slight obliquity which often prevents or delays the fœtus from freely entering the pelvis, and sometimes retards or "locks" it in its passage through it. I think the periænum is safer when upon the side, even if the labor is quicker, as the strong, voluntary pains, when upon the back, are most likely to cause rupture.

While the subject of position is important in nearly every case, its most evident triumphs are witnessed in some protracted and tedious cases. Did time and space permit, I could give a large number of cases in proof of what has been and can be done by position. A few illustrative ones are given, but as before observed, the application of the principle is very extensive, and must vary with every case:

Case I.—Mrs. P——. Was called in consultation, and this was one of the earlier cases to open my eyes in regard to position. Age 29; primipara in good health; been in labor three days; uterus dilated; vertex presentation; and pains strong. The cause of delay was not evident, but external examination showing some lateral obliquity, and viewing that as a possible cause, she was urged to lie upon the side to correct it. She said she could not lie upon the side desired, and would turn upon the back as soon as a pain commenced. Seeing this, we finally placed her upon the side desired, and kept her there during several pains, by one assistant at the shoulders and another at the hips. After about the second pain, the position was easy, and the case completed in a few hours, safely to mother and child. The proper position of the arm and the use of the abdominal bandage would have materially aided, if understood.

Case II.—Mrs. M——, age 30. Multipara large and healthy; been in labor two days. Was sent for with view of instrumental delivery. Head presentation; uterus dilated; pains strong. Diagnosis, as cause of delay, lateral and posterior obliquity. Position on one side, under arm flexed, and the use of bandage and support to back. Completed the labor successfully to mother and child within one hour.

Case III.—Mrs. S——, age 31. Multipara healthy; been in labor two days. Lateral obliquity, and been kept in wrong position; strength greatly exhausted; head presentation; uterus dilated, corrected position, after which labor advanced slowly, owing to the strength, when it was completed by forceps, safely to mother and child.

Case IV.—Mrs. H——, age 34. Multipara health imperfect; in labor six hours; uterus dilatable; head presenting; pains weak. Diagnosis, inertia. Nothing seemed to do any good until, with great difficulty, she was made to stand upon her feet, supported at the shoulders. Immediately the pains were strong, and from choice or obstinacy, she would not lie down again; the second pain, within ten minutes, completing the labor.

Case V.—Mrs. K——, aged 40. Multipara health good. In labor six hours; pains fair; uterus dilatable; head presentation. A bad case of placenta previa. Diagnosis, lateral obliquity. Placed on side, with arm back; the second pain, within five minutes, completing labor safely to mother and child.

Case VI.—Mrs. N——, aged 17. Multipara healthy; in labor seven hours; pains good; uterus so high could not diganose. After eight hours, found the head was resting upon the symphysis pubis. Placed upon the knees, on the floor and leaning far forward, the head just resting upon the side of the bed, and result was the successful completion of the case, within a few minutes.

Case VII.—Mrs. McG——, aged 35. Multipara health fair; in labor ten hours; pains irregular, uterus dilated; funis prolapsed, the left foot and breech presenting. Replaced the cord and held it a time with the hand. Delay partly owing to lateral obliquity and pendulous abdomen. Tried the side position with bandage, but complained of position, and was no advancement, except to correct the collateral obliquity. Then placed on back, folded a thick quilt, until some inches thick, and placed under the hips, the breech and position preventing the return of the cord. The whole head was kept wet with tepid water. After ten minutes, the quilt was removed, and immediately a strong continuous pain came on, which brought the foot and breech externally, while a few more shorter pains completed the labor, saving the child. The pulsation of the cord was carefully watched, and care was taken that the face was toward the sacrum.

After ten years of obstetric experience, I confess that I knew very little—next to nothing would be nearest the truth—of the true principles which should decide the particular position best for each case.

Neither do I think that my case was exceptional, for I have never met or known a practioner who had investigated the subject in that direction. It is even to be feared that the convenience of the accoucher, and not the woman's condition, has oftenest decided the position. While thus groping in the dark, how trying some cases were, and how the poor woman continued to suffer, and we could not tell why, or even help her! How we tried, perhaps vainly, to induce her to "change her position," and very properly too, as almost any change might prove a relief. What accoucher has not experienced more or less such cases, yet why they were so he could not discover; neither can any one tell in all cases.

Perhaps a physician's wife ought not to be a witness in her husband's case; but it is the opinion of my wife, that my obsteteric cases now average only about half the length of time that they formerly did.

I should feel to reproach myself for waiting so long since my attention was directed to the subject, before making it public, were not considerable time necessary for its satisfactory investigation.

Among the advantages sure to result from the future study of this subject, may be enumerated the following, viz:

1. Lessening the sufferings of the mother, and the risk to mother and child.
2. Shortening the duration of ordinary labor.
3. The necessity of instrumental delivery will often be avoided.
4. There will be a less number of "tedious," "powerless" and "obstructed" cases.
5. The community will be better able to discriminate between true science and pretension, in obsteteric practice.
6. The "curse" upon women will be partially removed.
7. Obsteteric practice will lose much of the dread which now usually attends it.

If I am too sanguine in my views and conclusions, the same are honestly expressed; and should they not all prove to be correct, I believe that they are in the direction of progress, and that what is now begun will be sure to be carried to greater completeness by future investigators.

The importance of the subject is my apology for the length of this communication.

---

HEMORRHOIDS, connected with prolapsus, were successfully operated upon by the application of nitric acid some thirty years ago by Dr. Houston of Dublin. The method found great favor at the time, but has been somewhat displaced by the ligature. Billroth, of Vienna, has of late revived Houston's operation, and reports excellent results.—*Canada Lancet.*

